# Diagnostic accuracy of transient elastography in MASLD is independent of MRI-PDFF steatosis in a multicenter study

**DOI:** 10.1016/j.jhepr.2025.101589

**Published:** 2025-09-11

**Authors:** Oumnia Masrour, Florent Ehrhard, Maeva Guillaume, Jerome Boursier, Jérôme Gournay, Karim Aziz, Matthieu Schnee, Ronan Garlantézec, Victor de Lédinghen, Jeff Morcet, Fabrice Lainé, Pierre Allaume, Bruno Turlin, Yves Gandon, Edouard Bardou-Jacquet

**Affiliations:** 1 Université de Rennes, CHU Rennes, Service des Maladies du Foie, F-35000 Rennes, France; 2 Service d’Hépatogastroentérologie, Centre Hospitalier Bretagne Sud, Lorient F-56100, France; 3 Clinique Pasteur, Toulouse F-31300, France; 4 CHU Angers, Service d’Hépatogastroentérologie, Angers F-49100, France; 5 CHU Nantes, Service d’Hépatogastroentérologie, Nantes F-44000, France; 6 Service d’Hépatogastroentérologie, Centre Hospitalier Yves Le Foll, Saint Brieuc F-22000, France; 7 Service d’Hépatogastroentérologie, CHD Vendée, La Roche sur Yon F-85000, France; 8 Université de Rennes, CHU Rennes, Département de Santé Publique, F-35000 Rennes, France; 9 CHU Bordeaux, Service d’Hépatogastroentérologie, Bordeaux F-33000, France; 10 Université de Rennes, CHU Rennes, Inserm, CIC 1414, F-35000 Rennes, France; 11 Université de Rennes, CHU Rennes, Service d’Anatomie et Cytologie Pathologiques, F-35000 Rennes, France; 12 Université de Rennes, CHU Rennes, Service de Radiologie et Imagerie Médicale, F-35000 Rennes, France; 13 Université de Rennes, CHU Rennes, Inrae, Inserm, NUMECAN – UMR_S 1317, F-35000 Rennes, France

**Keywords:** MASLD, MASH, MRI, PDFF, Transient elastography

## Abstract

**Background:**

Transient elastography (TE) can be used to accurately screen for severe liver fibrosis in metabolic dysfunction-associated steatotic liver disease (MASLD). However, some studies suggest that steatosis influences liver stiffness measurement (LSM). Thus, treatment that modifies steatosis might impact the relevance of TE in the management of patients. The use of magnetic resonance imaging (MRI)-proton density fat fraction (PDFF) has shown good reliability and accuracy for quantifying hepatic steatosis. Therefore, the aim of our study was to evaluate the performance of TE combined with MRI-PDFF for the diagnosis of severe fibrosis in patients with MASLD. The study is registered at ClinicalTrials.gov (NCT03245606).

**Methods:**

In this prospective multicenter study, patients with MASLD and indication for biopsy underwent MRI and TE within the same month. Biopsies were centrally reviewed using the steatosis, activity, and fibrosis (SAF) score. PDFF was centrally quantified using MRQuantif software. AUROCs for the diagnosis of fibrosis ≥F2 and ≥F3 were determined for LSM alone and LSM combined with PDFF as a continuous or categorical variable in a logistic regression.

**Results:**

In total, 208 patients were studied. The median age was 59 years, 63.9% were men, the median body mass index (BMI) was 31.2 kg/m^2^, and 47.6% had diabetes. Of these patients, 56.3% had fibrosis ≥F2 and 30.3% had fibrosis F3–F4 on biopsy. Based on the SAF score, 35.7% of patients had MASH. The AUROC of LSM for fibrosis ≥F3 was 0.78 (95% CI 0.72–0.85), with no significant difference compared with the AUROC of LSM combined with PDFF (0.78; 95% CI 0.72–0.85; *p* = 0.96). The AUROC of LSM for fibrosis ≥F2 was 0.67 (95 % CI 0.60–0.74), with no significant difference compared with the AUROC of LSM combined with PDFF (0.66; 95% CI 0.59–0.73, *p* = 0.48).

**Conclusions:**

TE performance for the diagnosis of liver fibrosis does not appear to be affected by the level of liver steatosis. Thus, quantifying hepatic steatosis using MRI-PDFF allows a comprehensive and accurate assessment of liver steatosis.

**Impact and implications:**

TE is a reliable tool for screening severe liver fibrosis in patients with MASLD. However, some studies suggest that the severity of steatosis influences LSM. Thus, treatment that modifies steatosis might impact the relevance of TE in the management of patients. This prospective multicenter study evaluated whether combining TE with MRI-PDFF, a precise method for measuring liver fat, could improve diagnostic accuracy for severe fibrosis in patients with MASLD. Our results show that TE performance in fibrosis diagnosis remains consistent regardless of steatosis levels, reinforcing the use of non-invasive methods, particularly TE, for regular patient monitoring, even as steatosis levels fluctuate.

## Highlights

• TE effectively screens for severe liver fibrosis in patients with MASLD.
• Existing data suggest that steatosis influences liver stiffness measurements, raising concerns about TE accuracy as steatosis levels fluctuate.
• This prospective multicenter shows that TE performance for fibrosis diagnosis is unaffected by steatosis levels as measured by MRI-PDFF, supporting TE as a robust, non-invasive monitoring tool even when steatosis levels change.

## Graphical abstract

**Figure:**
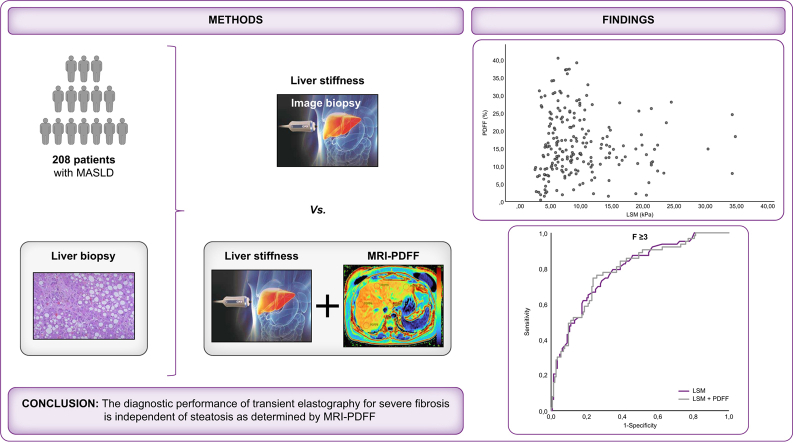

## Introduction

Metabolic dysfunction-associated steatotic liver disease (MASLD) is the leading cause of chronic liver disease worldwide, affecting 20–30% of the global population.^1^ Its prevalence is expected to increase along with rising rates of obesity, diabetes, and metabolic syndrome. It encompasses a wide spectrum of liver lesions, from benign hepatic steatosis or metabolic dysfunction-associated steatotic liver (MASL) to severe hepatocellular inflammation known as metabolic dysfunction-associated steatohepatitis (MASH).^2^ This latter condition confers a higher risk of progression to fibrosis, cirrhosis and hepatocellular carcinoma.^3^

The most relevant prognostic factor in the context of MASLD is the extent of liver fibrosis, which directly correlates with the long-term prognosis.^4^^,^^5^ Liver biopsy remains the gold standard for the diagnosis of fibrosis. However, its invasiveness, sampling variability, and costs make it unsuitable as a first-line diagnostic tool.

Recently, multiple non-invasive diagnostic methods have been investigated, notably transient elastography (TE), the performance of which has been validated for the diagnosis of advanced fibrosis.^6^^,^^7^ Nevertheless, some specific lesions associated with MASLD might affect the accuracy of this non-invasive liver stiffness measurement (LSM). For instance, histological steatosis appears to be associated with an overestimation of LSM, as confirmed in several studies on chronic HCV.^8^^,^^9^ In terms of MASLD, a large prospective study by Petta *et al.* suggests that steatosis is independently associated with higher LSM, which could hamper the diagnostic performance of TE.^10^ However, in this study, steatosis severity was determined by liver biopsy, thereby undermining the relevance of a non-invasive test. The influence of steatosis on LSM is crucial, particularly given that steatosis can evolve in response to the pharmacological treatment or progression of metabolic syndrome. This raises an important question regarding the significance of LSM variation over time, because it might reflect steatosis changes or evolution of liver fibrosis.

Liver magnetic resonance imaging (MRI) with proton density fat fraction (PDFF) sequence has demonstrated its reliability and precision in non-invasive diagnosis of steatosis.[11], [12], [13] The quantification of liver steatosis by MRI-PDFF is accessible, highly reproducible and sensitive, and offers the advantage of assessing the overall liver volume. Therefore, our study investigated the impact of steatosis quantified by MRI-PDFF on the performance of TE for diagnosing liver fibrosis in MASLD.

## Patients and methods

### Study design

This was a prospective, multicenter study conducted in eight secondary and tertiary care centers in France (Rennes University Hospital, Lorient Hospital, Toulouse University Hospital, Angers University Hospital, Nantes University Hospital, Saint Brieuc Hospital, La Roche Sur Yon Hospital, and Bordeaux University Hospital).

Between January 2018 and March 2020, all patients with clinical indication of liver biopsy for suspected MASLD, as determined by their referring hepatologist, were consecutively enrolled. Enrolled patients were invited to undergo standardized history, physical and anthropometric evaluation, laboratory testing, MRI, and TE.

All patients provided informed written consent before enrolling in the study. The study was conducted in accordance with the Declaration of Helsinki and was approved by the Research Ethics Committee (CPP Sud Méditerranée 5 17.028). This study is registered on Clinicaltrials.gov as NCT03245606. STARD guidelines were used to report the protocol.^14^

### Inclusion and exclusion criteria

Adults (≥18 years) undergoing a liver biopsy for suspected MASLD, presenting with at least one of the criteria of metabolic syndrome as defined by the International Diabetes Federation, were included in the study (*i.e.* body mass index [BMI] >25 kg/m^2^ and/or waist circumference ≥94 cm in men and ≥80 cm in women; fasting plasma glucose ≥5.6 mmol/L or previously diagnosed type 2 diabetes mellitus; blood pressure ≥130/85 mmHg or treatment of previously diagnosed hypertension; triglycerides ≥1.7 mmol/L; HDL cholesterol ≤1 mmol/L in men and ≤1.3 mmol/L in women).^15^

Exclusion criteria were: alcohol intake ≥210 g/week in men or ≥140 g/week in women; non-MASLD liver disease (including chronic HBV or HCV, hemochromatosis, Wilson’s disease, autoimmune hepatitis, primary sclerosing cholangitis, primary biliary cholangitis, α1-antitrypsin deficiency, or other causes of chronic liver disease); secondary cause of hepatic steatosis (medication that can cause steatosis: corticosteroids, amiodarone, methotrexate, or tamoxifen); history of bariatric surgery; or contraindications to MRI.

### Clinical evaluation

All patients underwent a standardized clinical evaluation, which included medical history, anthropometric assessment, and biochemical tests. Information from the medical history and anthropometric exam included age, sex, height, weight, BMI, vital signs, and assessment of alcohol intake history. Biochemical tests included fasting glucose, hemoglobin A1c, insulin, triglycerides, total cholesterol, HDL, LDL, ferritin, aspartate aminotransferase (AST), alanine aminotransferase (ALT), alkaline phosphatase (ALP), gamma glutamyl transferase (GGT), total bilirubin, albumin, prothrombin time, international normalized ratio, and platelet count.

### Transient elastography

TE was performed using the Fibroscan® 502 Touch model (Echosens, Paris, France) in each center by a trained nurse, who was blinded to the patient’s clinical data and histological evaluation. Following the manufacturer’s recommendations, the M probe was used for patients with BMI <30 kg/m^2^, and the XL probe for patients with BMI ≥30 kg/m^2^. At least 10 measurements were made to obtain the median valid LSM in kilopascals (kPa) and the IQR. LSM was considered valid if the IQR/median was <30% if the LSM was >7.1 kPa.

### MRI proton density fat fraction

MR studies were obtained from eight different MRI scanners, four with a 3T magnetic field (Signa Pioneer [GE Healthcare, Chicago, IL, USA], Ingenia [Philips, Amsterdam, The Netherlands], Prisma or Skyra [Siemens, Erlangen , Germany]), and four with a 1.5T field (Achieva or Ingenia [Philips], and Aera or Avanto [Siemens]). Radiologists were blinded to the patient’s clinical data and histological evaluation.

Liver MRI was performed at the time of biopsy or within a maximum of 1 month. Each MR examination included a non-contrast, breathhold chemical shift-encoded (CSE) gradient recalled-echo (GRE) 2D axial sequence. A minimum of three slices, each 7–10 mm thick, was obtained from the middle part of the liver. The parameters were defined according to the guidelines proposed on MRQuantif.org. PDFF values were calculated centrally by an experienced reader (YG), using the MRQuantif software (Université de Rennes, Rennes, france), validated against biopsy.^16^

### Liver histology

Percutaneous liver biopsies were performed according to local standard procedure (supplementary data). Slides were analyzed in each center as part of standard care and then centrally reviewed by a single senior expert liver pathologist (BT) blinded to clinical information and to TE and MRI data.

Biopsies were assessed using the SAF scoring system,^17^ in which steatosis was graded as follows: 0, <5%; 1, 6–33%; 2, 34–66%; and 3, >66%. Activity grade was based on the presence of hepatic ballooning scored from 0 to 2; and lobular inflammation scored from 0 to 2. Hepatic fibrosis was scored on a scale from 0 to 4: stage 0, absence of fibrosis; stage 1, perisinusoidal or portal; stage 2, perisinusoidal and portal/periportal; stage 3, septal or bridging fibrosis; and stage 4, cirrhosis. MASH was defined by the presence of steatosis in >5% of hepatocytes, the presence of hepatocellular ballooning of any degree, and lobular inflammation of any amount.

### Outcomes

The primary outcome was the diagnosis of fibrosis stage ≥3. The secondary outcomes were: the diagnosis of fibrosis stage ≥2 and the diagnosis of fibrotic MASH (defined by the presence of MASH as defined above and fibrosis stage ≥2).

### Statistical analysis

#### Sample size calculation

We hypothesized an increase in the AUROCs from 0.79 [18] to 0.88 [10] (corresponding to the AUROC in the absence of steatosis). Based on literature data and the experience of the participating centers, we estimated the prevalence of advanced fibrosis to be 25%. The correlation of diagnostic tests in patients positive for advanced fibrosis was estimated to be 0.6, and in patients negative for advanced fibrosis to be 0.8. To demonstrate a significant difference between the ROC curves, with an alpha risk of 5% and a beta of 20%, the required sample size was 216. Considering a typical failure rate of 10% for TE, we estimated that 240 participants would be needed.

#### Descriptive statistics

Continuous variables were summarized as median (IQR) and categorical variables as frequency and percentage. The Mann-Whitney *U* test, Kruskal-Wallis test and Chi-square test were used as appropriate. The Spearman correlation rank test was used to assess correlations between continuous or ordinal variables. All analyses were performed using IBM SPSS statistics version 26 (IBM Corp., College Station, Armonk, New York, USA); *p* <0.05 was considered statistically significant.

#### Combination of LSM and PDFF

The weighted combination of LSM and PDFF was computed using a multivariate logistic regression. The dependent variable comprised the diagnosis of liver fibrosis (fibrosis stage ≥3) as determined by liver biopsy. LSM assessed by TE was introduced as an explanatory variable while adjusting for PDFF, expressed as a qualitative (≤3.5%; 3.6–14.8%; 14.9–21.7%; >21.7%) or continuous variable.

#### AUROC comparison

Performances were assessed using AUROCs with 95% CIs for LSM alone and for the predictive equation combining LSM and PDFF. AUROCs were compared using the DeLong test.^19^ The cut-off value was determined using Youden’s index. Sensitivity (Se), specificity (Sp), positive predictive value (PPV), and negative predictive value (NPV) were also assessed.

#### Sensitivity analysis

Additional analyses were conducted to assess the AUROCs for LSM and LSM combined with PDFF across subgroups based on BMI (≥30 *vs.* <30 kg/m^2^) and the presence of diabetes.

## Results

### Study population

**Fig. 1:**
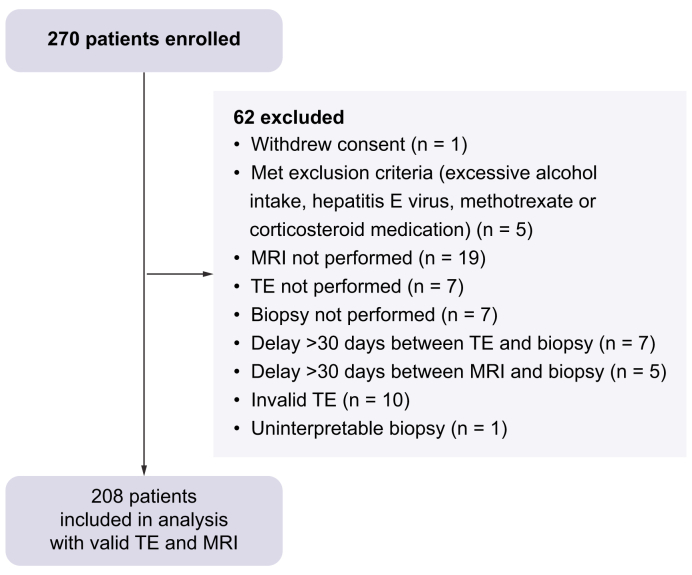
Study flow chart. |MRI, magnetic resonance imaging; TE, transient elastography.

A total of 270 patients were included initially, 62 of whom were excluded because of the absence of TE or MRI, a delay >30 days, or uninterpretable TE (Fig. 1). The failure rate of TE was 4% (10 invalid TEs out of 250 examinations performed). The resulting studied population comprised 208 patients, the baseline cohort characteristics of which are detailed in Table 1. Participants were predominantly men (63.9%), with a median age of 59 (51–65) years. The median BMI was 31.2 (28.7–35.5) kg/m^2^.

**Table 1:** Patient characteristics.

Characteristic	Patients (n = 208)
**Demographics**	
Male, n (%)	133 (63.9)
Age (years), median (IQR)	59 (51–65)
BMI (kg/m^2^), median (IQR)	31.2 (28.7–35.5)
BMI ≥30 kg/m^2^, n (%)	128 (61.5)
Hypertension, n (%)	120 (57.7)
Diabetes, n (%)	99 (47.6)
Hyperlipidemia, n (%)	124 (59.6)

**Biochemical profile**	
AST (IU/L), median (IQR)	37 (25.5–56)
ALT (IU/L), median (IQR)	51 (32–85.25)
ALP (IU/L), median (IQR)	72 (60.25–93)
GGT (IU/L), median (IQR)	67 (39–126)
Total bilirubin (mmol/L), median (IQR)	9 (7–12)
Albumin (g/L), median (IQR)	43.3 (41–41.95)
Hemoglobin A1c (%), median (IQR)	6.1 (5.6–7.35)
Triglycerides (mmol/L), median (IQR)	1.65 (1.17–2.29)
Total cholesterol (mmol/L), median (IQR)	4.8 (4–5.59)
HDL (mmol/L), median (IQR)	1.11 (0.96–1.32)
LDL (mmol/L), median (IQR)	2.66 (2.05–3.54)
Platelet count (10^9^/L), median (IQR)	219 (184–258)

**Histology**	
Steatosis grade, n (%)	
0	13 (6.2)
1	91 (43.8)
2	62 (29.8)
3	42 (20.2)
Lobular inflammation grade, n (%)	
0	52 (25)
1	129 (62)
2	27 (13)
Ballooning grade, n (%)	
0	134 (64.4)
1	53 (25.5)
2	21 (10.1)
Fibrosis stage, n (%)	
0	16 (7.7)
1	75 (36.1)
2	54 (26)
3	26 (12.5)
4	37 (17.7)
Presence of MASH, n (%)	74 (35.7)
Presence of fibrotic MASH, n (%)	57 (27.4)

**Imaging**	
Liver stiffness (kPa), median (IQR)	8.2 (5.6–11.6)
Liver stiffness categories, n (%)	
≤8 kPa	101 (48.6)
8.1–12 kPa	59 (28.4)
>12 kPa	48 (23.1)
MRI-PDFF (%), median (IQR)	14.8 (9.8–22.0)
MRI-PDFF categories, n (%)	
≤3.5%	15 (7.2)
3.6–14.8%	90 (43.3)
14.9–21.7%	51 (24.5)
≥21.8%	52 (25)

ALP, alkaline phosphatase; ALT, alanine aminotransferase; AST, aspartate aminotransferase; BMI, body mass index; GGT, gamma glutamyl transferase; MASH, metabolic dysfunction-associated steatohepatitis; MRI, magnetic resonance imaging; PDFF, proton density fat fraction.

TE was performed on the same day as the biopsy. The median interval between MRI and biopsy was 3 (0–2) days.

Of the patients, 13 (6.2%), 91 (43.8%), 62 (29.8%), and 42 (20.2%) had grade 0, 1, 2, and 3 steatosis, respectively; and 16 (7.7%), 75 (36.1%), 54 (26%), 26 (12.5%), and 37 (17.7%) had stage 0, 1, 2, 3, and 4 fibrosis, respectively. Furthermore, 107 (51.4%) patients had LSM >8 kPa and 47 (22.6%) had LSM >12 kPa. According to MRI-PDFF with cut-offs 3.5%, 14.8%, and 21.7%, 90 (43.3%), 51 (24.5%) and 52 (25%) had grade 1, 2, and 3 steatosis, respectively.

### Fibrosis is associated with histological steatosis grade but not with MRI-PDFF

The median PDFF was 14.8% (9.8–22; Fig. S1). For the detection of steatosis grade ≥1, the AUROC for PDFF was 0.98 (95% CI 0.96–1).

There was a significant correlation between the stage of fibrosis and the histological grade of steatosis (rho = 0.15, *p* = 0.039) but not with PDFF regardless of whether treated as an ordinal or continuous variable (rho = 0.035, *p* = 0.62).

**Fig. 2:**
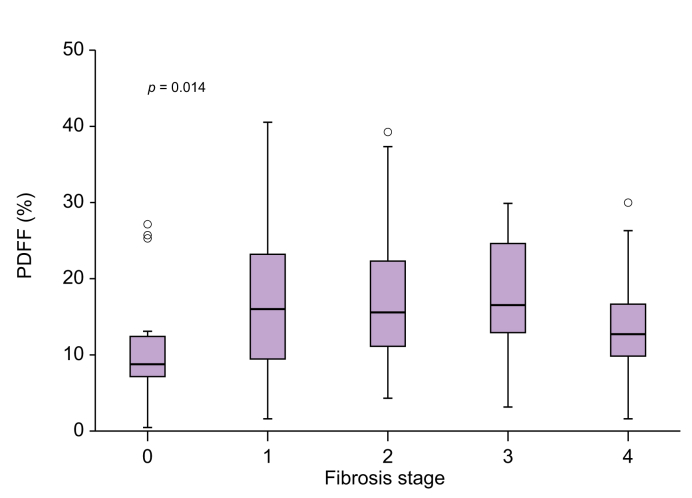
Distribution of steatosis measurements by MRI-PDFF stratified by fibrosis stage. PDFF measurements were lower in the F0 and F4 groups. Data are presented as median + interquartile range and analyzed with the Kruskal-Wallis test, *p* = 0.014). MRI, magnetic resonance imaging; PDFF, proton density fat fraction.

There was a significant difference in the distribution of PDFF across fibrosis stages (Kruskal-Wallis test, *p* = 0.014; Fig. 2): PDFF measurements were significantly lower in the F0 and F4 groups compared with the F1, F2, and F3 groups, where PDFF values were comparable. A similar trend was observed when analyzing steatosis grade across fibrosis stages (Fig. S2).

### LSM shows high diagnostic accuracy performance for detecting fibrosis stage ≥F3

**Fig. 3:**
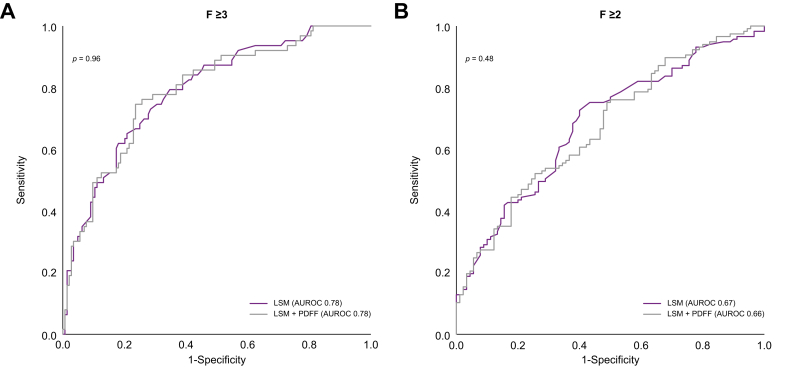
Diagnostic performance of LSM and LSM + PDFF for (A) fibrosis stage ≥F3 (DeLong test, *p* = 0.96) and (B) fibrosis stage ≥F2 (DeLong test, *p* = 0.48). LSM, liver stiffness measurement; PDFF, proton density fat fraction.

The median LSM was 8.2 kPa (Fig. S3). The AUROC of LSM for diagnosing fibrosis stage ≥3 was 0.78 (0.72–0.8)] (Fig. 3A), whereas that of LSM for diagnosing fibrosis stage ≥2 was 0.67 (0.60–0.74) (Fig. 3B).

Cut-off values of 8 kPa and 12 kPa had a sensitivity of 79.4% and a specificity of 88.3% to rule out and rule in fibrosis stage ≥3, respectively (Table S1).

### LSM values are unaffected by PDFF across fibrosis stages

There was no significant difference in the distribution of LSM across histological steatosis grades (Fig. S4). This finding remained consistent after stratifying the analysis by fibrosis stage (Figs. S5 and S6).

Similarly, there was no significant correlation between LSM and PDFF (rho = 0.04, *p* = 0.56).

**Fig. 4:**
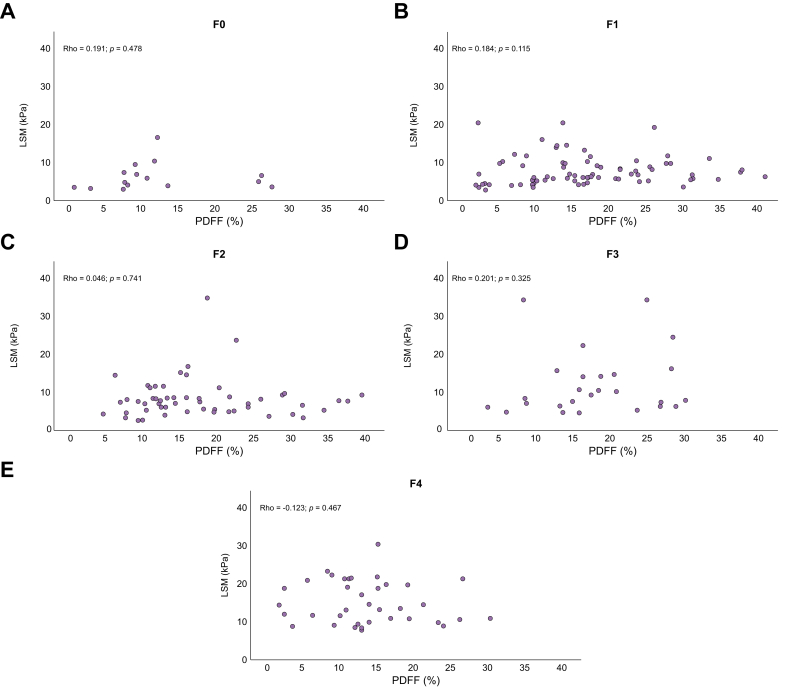
LSM values distribution according to PDFF, within patients with fibrosis stage (A) 0, (B) 1, (C) 2, (D) 3, and (E) 4. Analysis based on Spearman correlation rank test. LSM, liver stiffness measurement; PDFF, proton density fat fraction.

Assessment of the variations in LSM values according to PDFF across the different fibrosis stages (Fig. 4) showed that, within each fibrosis stage, LSM values remained consistent, independently of PDFF levels.

### Diagnostic performance of LSM is not enhanced by accounting for MRI-PDFF

We investigated the diagnostic accuracy of the score derived from the predictive equation integrating LSM and PDFF, as described above. The AUROC for diagnosing fibrosis stage ≥3 was 0.78 (0.72–0.85) for LSM + PDFF as a continuous variable, similar to LSM alone (DeLong test, *p* = 0.96; Fig. 3A).

**Fig. 5:**
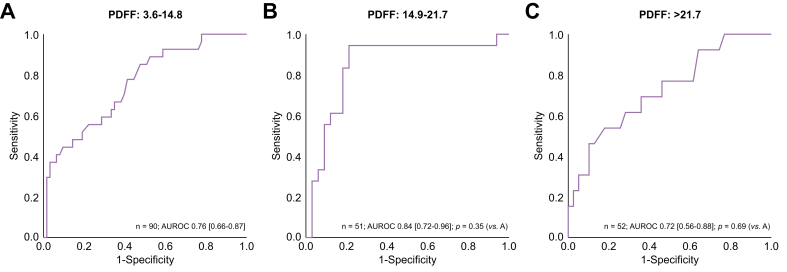
AUROCs of LSM for diagnosing fibrosis stage ≥3 across PDFF categories. AUROCs were compared with the reference group PDFF 3.6–14.8% using the DeLong test: (A) PDFF 3.6–14.8%; (B) PDFF 14.9–21.7% (*p* = 0.35); and (C) PDFF >21.7% (*p* = 0.69). LSM, liver stiffness measurement; PDFF, proton density fat fraction.

**Fig. 6:**
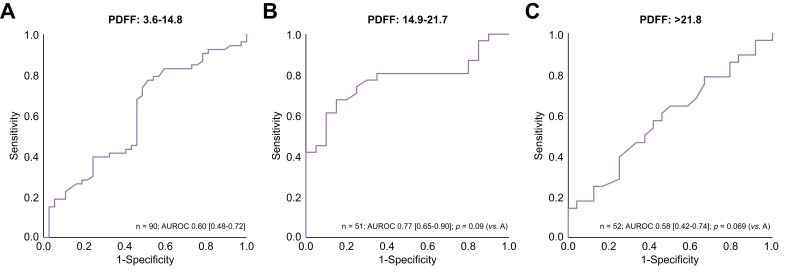
AUROCs of LSM for diagnosing fibrosis stage ≥2 across PDFF categories. AUROCs were compared with the reference group PDFF 3.6–14.8% using the DeLong test: (A) PDFF 3.6–14.8%. (B) PDFF 14.9–21.7% (*p* = 0.09); and (C) PDFF >21.7% (*p* = 0.069). LSM, liver stiffness measurement; PDFF, proton density fat fraction.

The AUROC for diagnosing fibrosis stage ≥2 was 0.66 (0.59–0.73) for LSM + PDFF as a continuous variable (*p* = 0.48; Fig. 3B). The AUROCs of LSM for diagnosing fibrosis stage ≥3 and ≥2 were comparable across steatosis categories as determined by PDFF (Fig. 5, Fig. 6). The AUROC for diagnosing fibrotic MASH was 0.73 (0.66–0.81) for both LSM and LSM + PDFF (*p* = 0.93). The cut-off levels, Se, Sp, PPV, and NPV for each fibrosis stage are detailed in Table 2.

**Table 2:** Diagnostic accuracy of LSM and LSM + PDFF for each stage of fibrosis.

Fibrosisstage	LSM							LSM + PDFF							*p* value
	AUROC	95% CI	Cut-off	Se	Sp	PPV	NPV	AUROC	95% CI	Cut-off	Se	Sp	PPV	NPV	
≥1	0.68	0.53–0.84	5.1	83.9	50	95.3	20.5	0.69	0.55–0.84	0.91	70.3	75	97.1	16.9	0.93
≥2	0.67	0.60–0.74	7	72.7	59.3	69.7	62.8	0.66	0.59–0.73	0.51	75.2	50.6	65.6	59.7	0.48
≥3	0.78	0.72–0.85	8.3	79.4	64.8	49.5	87.9	0.78	0.72–0.85	0.27	74.6	75.9	57.3	87.3	0.96
4	0.84	0.79–0.90	8.4	97.3	62	35.6	99.1	0.81	0.74–0.88	0.16	81.1	70.8	36.7	93.8	0.21
Fibrotic MASH	0.73	0.66–0.81	7.3	86	53	40.8	90.9	0.73	0.66–0.81	0.27	67	72	45.8	84.8	0.93

*p* values are those of the comparison of AUROCs using the DeLong test. LSM, liver stiffness measurement; MASH, metabolic dysfunction-associated steatohepatitis; NPV, negative predictive value; PDFF, proton density fat fraction; PPV, positive predictive value; Se, sensitivity; Sp, specificity.

### Sensitivity analyses

Subgroup analyses yielded similar results across subgroups defined according to BMI or status with respect to type 2 diabetes mellitus. AUROCs of LSM and LSM + PDFF for diagnosing fibrosis stages and fibrotic MASH did not differ between participants with or without type 2 diabetes mellitus (Table S2) or between patients with BMI ≥30 kg/m^2^ and those with BMI <30 kg/m^2^ (Table S3).

To evaluate the impact of liver inflammation, exploratory analyses incorporating ALT into LSM or LSM + PDFF models did not improve diagnostic performance for fibrosis staging. Similar results were observed when using the presence of histological MASH instead of ALT.

### CAP is associated with steatosis and does not improve LSM performance

There was a significant correlation between CAP values and histological steatosis (rho = 0.495; *p* <0.001), and between PDFF and CAP (rho = 0.534; *p* <0.001).

We performed an exploratory analysis to assess the impact of CAP values on the diagnostic performance of LSM, notwithstanding the substantial amount of missing data. AUROCs for diagnosing fibrosis stages ≥3, ≥2, and fibrotic MASH were similar for LSM + CAP and LSM alone (Table S4 and Fig. S7).

## Discussion

To our knowledge, our study is the first to directly compare LSM measured using TE and the combination of LSM and MRI-PDFF, in a prospective, large, multicenter, well-characterized population of patients with MASLD using liver biopsy as a reference. Our findings show that LSM is not associated with the level of steatosis as determined by MRI-PDFF, thus indicating that the diagnostic performance of TE for severe liver fibrosis is independent of steatosis.

Non-invasive tests for assessing liver fibrosis assessment have become increasingly popular over the past decade as alternatives to liver biopsy, driving major practice changes in hepatology.^20^ TE is a simple and reliable non-invasive tool to assess liver fibrosis in various chronic liver diseases, including MASLD. Indeed, Wong *et al.* reported AUROCs of 0.84 for diagnosing significant fibrosis and 0.93 for advanced fibrosis in a 2010 study on patients with MASLD.^6^ Similarly, a recent meta-analysis reported high diagnostic accuracy of TE in patients with MASLD with AUROCs of 0.83 and 0.85 for diagnosing significant and advanced fibrosis, respectively.^7^ In our study, the AUROCs for LSM were slightly lower. This discrepancy might be attributed to population characteristics, given the higher median BMI in our study, which could affect the performance of TE. In addition, it might be related to inherent interoperator variability with TE given that our study is multicentric and includes secondary centers, whereas most studies to date have been performed in large tertiary centers.

In our study, MRI-PDFF was used to quantify steatosis. This approach is simple to use and accessible even in non-expert centers. There was a high correlation between MRI-PDFF and histological steatosis, demonstrating its excellent performance for the detection of steatosis. Our findings are consistent with existing literature on the accuracy and reliability of MRI-PDFF as a non-invasive biomarker of steatosis.[11], [12], [13]

We compared the diagnostic performance of LSM combined with PDFF and LSM alone for fibrosis diagnosis. Our results showed that incorporating steatosis into the assessment did not impact the accuracy of LSM.

Several studies have examined factors that can affect TE performance, with a particular focus on histological lesions associated with MASLD, including steatosis. Higher LSM values in the presence of liver steatosis have been reported in patients with chronic HCV,^8^^,^^9^ but data are controversial regarding MASLD. Indeed, although several studies did not identify an association between steatosis and LSM,^6^^,^^21^ Petta *et al.* reported the independent association between histological steatosis and a higher LSM measured by TE in a large study conducted in 2015,^10^ leading them to propose different LSM thresholds based on steatosis grade. These findings were later confirmed, with steatosis measured by controlled attenuation parameter (CAP) rather than biopsy.^22^ However, all the patients in their cohort underwent TE with the M probe despite an elevated BMI, which could have led to an overestimation of LSM. In addition, the higher LSM observed in the severe steatosis group might be attributed to a greater proportion of patients with fibrosis stage ≥2 in this subgroup. Finally, despite a strong correlation, the information on steatosis provided by histology and MRI-PDFF differs slightly, given that histology indicates the proportion of hepatocytes that are macroscopically fatty, whereas PDFF estimates the overall volume fraction of lipids in the liver.^23^

Our findings could have important implications for the future management of patients undergoing treatments that impact steatosis. Indeed, current therapeutic management of MASLD primarily focuses on lifestyle and dietary changes aimed at achieving a 7–10% weight loss, which is known to improve steatosis.^24^ Meanwhile, research into pharmacological treatments for MASLD is rapidly advancing, with most therapies leading to a reduction in steatosis. For instance, resmetirom, a thyroid hormone receptor beta-selective agonist that recently received conditional FDA approval, exhibited a reduction of 35–46% in steatosis, as assessed by MRI-PDFF.^25^ These developments raise important considerations regarding both patient follow-up and treatment efficacy evaluation. One might question whether the evolution of LSM during follow-up merely reflects steatosis resolution or indicates actual improvement in liver fibrosis. Our cross-sectional findings indicate that LSM values are not influenced by steatosis as measured by MRI-PDFF across the wide spectrum observed in our population, suggesting that the diagnostic performance of LSM is not impacted by concurrent steatosis levels. Although this implies that longitudinal changes in steatosis do not impact LSM over time, our cross-sectional design does not allow us to definitively confirm this hypothesis, which would require longitudinal follow-up data. This supports the parallel use of LSM and MRI-PDFF as non-invasive tools for monitoring patients over time. Alternative techniques for monitoring steatosis are available, such as CAP, but these methods lack the sensitivity and linearity of MRI-PDFF, limiting their utility for accurate longitudinal assessment. This underscores the need for continued research to improve and validate accessible, precise tools for tracking dynamic changes in liver fat content over time. Practically, MRI-PDFF could be considered useful in parallel to TE for providing follow-up information regarding short-term treatment or lifestyle modification effects on liver steatosis level, while TE offers more long-term follow-up information regarding liver fibrosis.

Our study has several strengths: first, it is a prospective multicenter study derived from a well-characterized cohort of patients with MASLD. Second, it involved not only tertiary centers, but also non-expert centers, highlighting the accessibility and widespread applicability of TE and MRI-PDFF. This broad inclusion enhances the generalizability of the results. Third, liver fibrosis diagnosis was based on liver biopsy, centrally reviewed by a single expert pathologist. Finally, to our knowledge, this is the only study to compare head-to-head LSM to the combination of LSM and PDFF.

However, our study has limitations that should be acknowledged. First, the number of patients ultimately included in the analysis was slightly lower than the calculated sample size estimate, which might have hampered the statistical power of the study. Second, liver biopsy indication was not standardized between the centers, and was ultimately left to physician discretion. Although this might have introduced some heterogeneity, it reflects real-world practice. Third, liver biopsy, although considered the gold standard for assessing liver pathology, has limitations related to sampling errors as well as intra- and interobserver variability.^26^ Fourth, slides were centrally reviewed by a single senior expert liver pathologist blinded to clinical data; although this approach ensured consistency, the absence of a second independent review might have introduced bias. Finally, we acknowledge the lack of follow-up data in the design of our study; longitudinal studies would be valuable for obtaining follow-up data and assessing the prognostic value of these variations.

In conclusion, quantifying hepatic steatosis using MRI-PDFF provides a comprehensive and accurate assessment of liver steatosis. In addition, TE performance for diagnosing liver fibrosis is not affected by the degree of liver steatosis measured by MRI-PDFF.

## Abbreviations

ALP, alkaline phosphatase; ALT, alanine aminotransferase; AST, aspartate aminotransferase; BMI, body mass index; CAP, controlled attenuation parameter; CSE, chemical shift-encoded; GGT, gamma glutamyl transferase; GRE, gradient recalled-echo; LSM, liver stiffness measurement; MASH, metabolic dysfunction-associated steatohepatitis; MASL, metabolic dysfunction-associated steatotic liver; MASLD, metabolic dysfunction-associated steatotic liver disease; MRI, magnetic resonance imaging; NPV, negative predictive value; PDFF, proton density fat fraction; PPV, positive predictive value; SAF, steatosis, activity, and fibrosis; Se, sensitivity; Sp, specificity; TE, transient elastography.

## Financial support

This study was supported by a grant from the French 10.13039/100009647Ministry of Health (PHRC 2016, PHRCI-16-054).

## Authors’ contributions

Conceptualization: EBJ, OM, YG, BT. Formal analysis: EBJ, OM, JM. Funding acquisition: EBJ. Investigation: FE, MG, JB, JG, KA, MS, VL, FL, PA, BT, YG, EBJ, OM. Methodology: EBJ, OM, RG. Validation and visualization: EBJ, OM. Writing – original draft: OM, EBJ. Writing – review and editing: all authors.

## Data availability statement

The datasets analyzed during this study are available from the corresponding author upon reasonable request.

## Conflicts of interest

The authors declare no conflicts of interest.

Please refer to the accompanying ICMJE disclosure forms for further details.
