# Supplementary material for: Diagnostic accuracy of transient elastography in MASLD is independent of MRI-PDFF steatosis in a multicenter study
Source: JHEP Rep. 2025 Sep 11;7(12):101589. doi: 10.1016/j.jhepr.2025.101589 (PMC12666573; doi:10.1016/j.jhepr.2025.101589)
Supplement: Multimedia component 1 [file mmc1.pdf]

Diagnostic accuracy of transient elastography in MASLD is independent of MRI-PDFF steatosis in a multicenter study

Oumnia Masrour, Florent Ehrhard, Maeva Guillaume, Jerome Boursier, Jérôme Gournay, Karim Aziz, Matthieu Schnee, Ronan Garlantézec, Victor de Lédinghen, Jeff Morcet, Fabrice Lainé, Pierre Allaume, Bruno Turlin, Yves Gandon, Edouard Bardou-Jacquet

Table of contents

Standard Procedure for Percutaneous Liver Biopsy……………………………………………..2

Fig. S1……………………………………………………………………………………..3

Fig. S2……………………………………………………………………………………..3

Fig. S3……………………………………………………………………………………..4

Fig. S4……………………………………………………………………………………..4

Fig. S5……………………………………………………………………………………..5

Fig. S6……………………………………………………………………………………..6

Fig. S7……………………………………………………………………………………..7

Table S1…………………………………………………………………………………..8

Table S2…………………………………………………………………………………..8

Table S3…………………………………………………………………………………..8

Table S4…………………………………………………………………………………..9

Standard Procedure for Percutaneous Liver Biopsy

Informed consent was obtained after explaining the procedure, risks, and benefits.

Pre-procedure assessment included coagulation profile, platelet count: the following cut-off are used: prothrombin time >50%, platelet count >60G/L, activated partial thromboplastin time ratio <1.4

Anticoagulant therapy was interrupted one week before biopsy, with replacement by low molecular weight heparin if required.

Biopsy must be guided by ultrasound examination.

Patients were instructed to fast for at least 6 hours prior to the biopsy.

Biopsy proceedings:

The patient was positioned in the left lateral decubitus position.

Ultrasound guidance is performed to identify an appropriate biopsy site in the right hepatic lobe.

The skin at the puncture site was carefully disinfected twice using an antiseptic solution (e.g., chlorhexidine)

Sterile drapes were placed to maintain an aseptic field around puncture site

Local anesthesia was administered at the skin puncture site.

Then a 16-gauge biopsy needle (Menghini or TruCut) was used to obtain a liver tissue sample.

Patients were monitored for at least 6 hours after the biopsy.

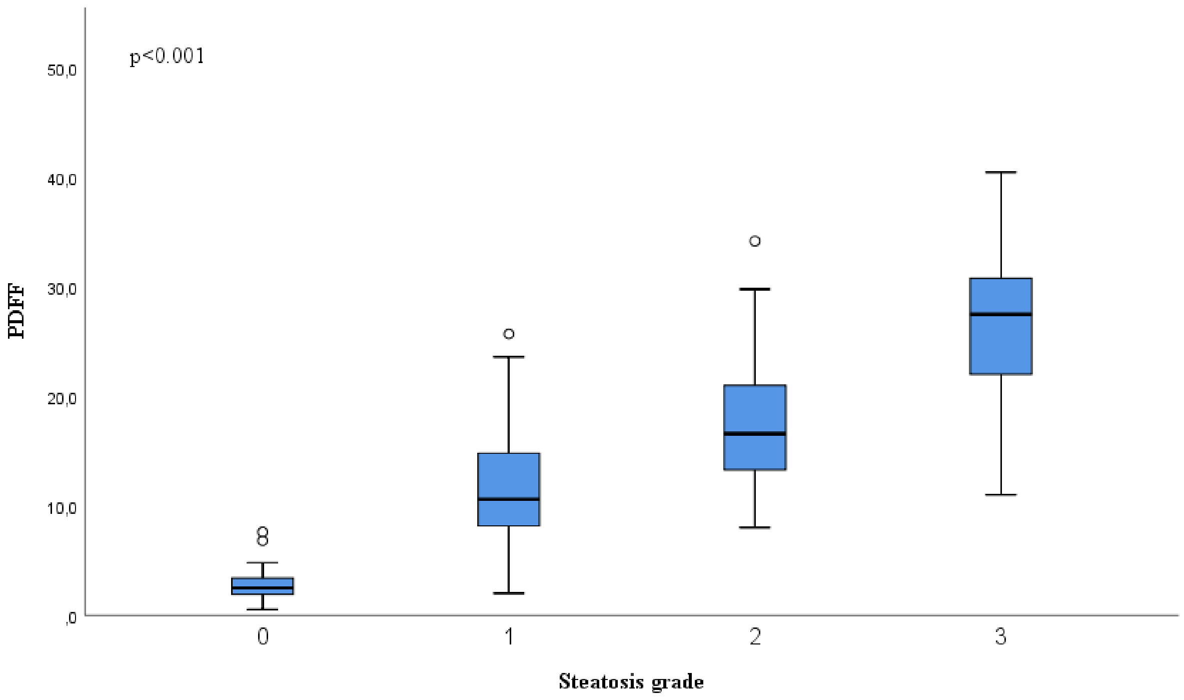

Fig. S1: Distribution of steatosis measurements by MRI-PDFF across steatosis grades. (p<0.001; Kruskall-Wallis test)

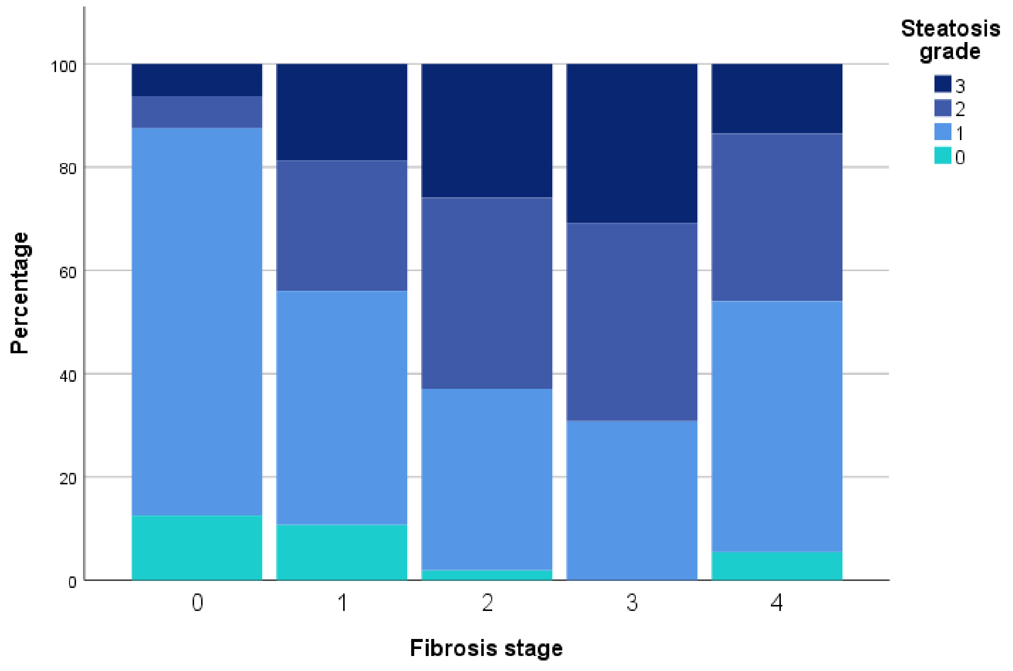

Fig. S2: Distribution of histological steatosis grades across fibrosis stages ; (p=0.036; Chi squared test)

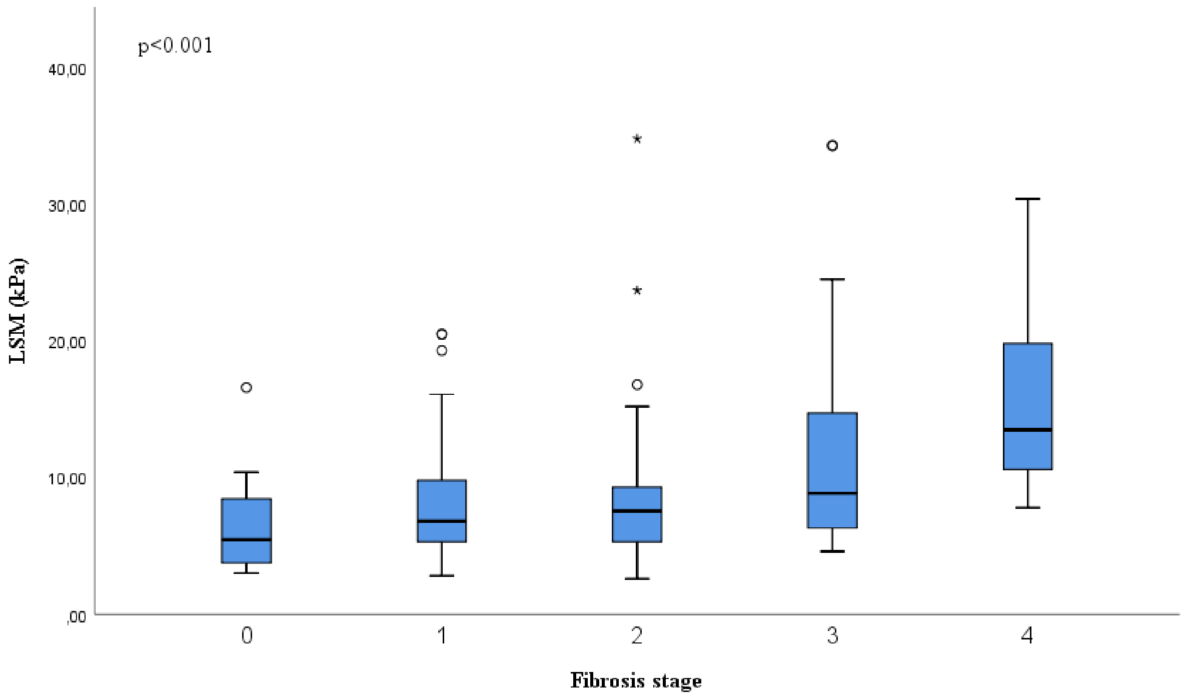

Fig. S3: Distribution of LSM measurements across histological fibrosis stages. ($p<0.001$; Kruskall-Wallis test)

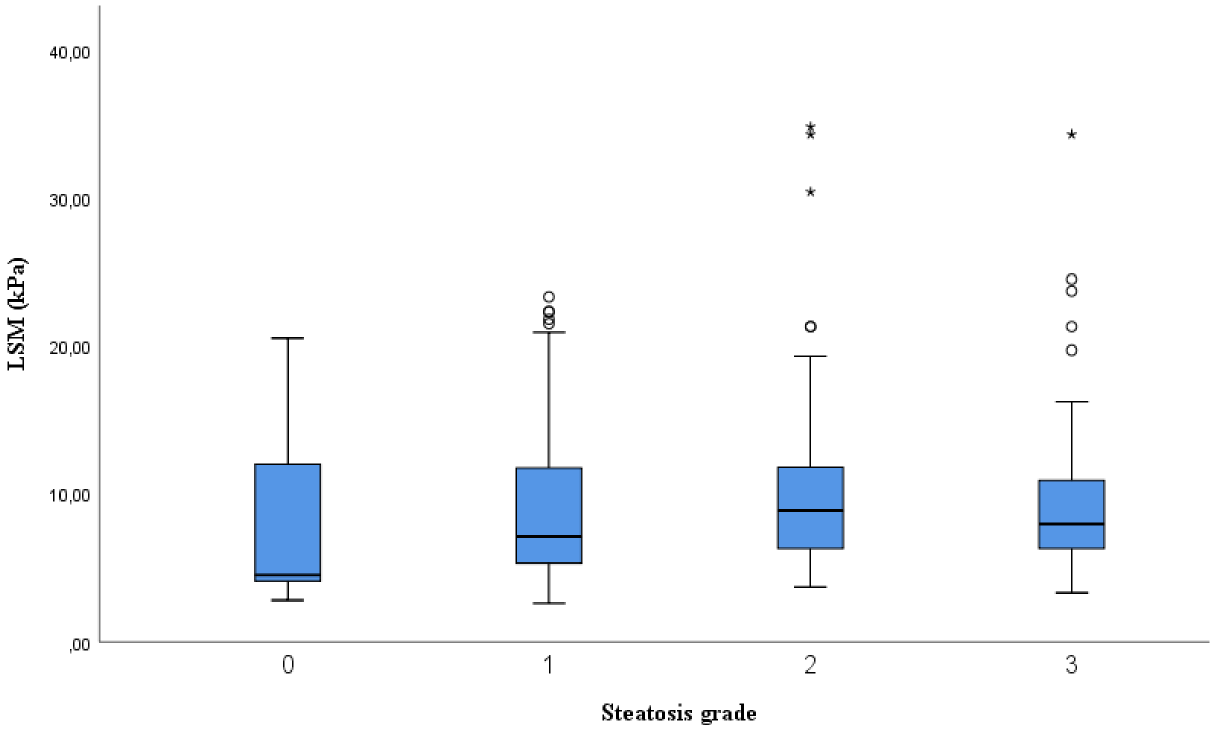

Fig. S4: Distribution of LSM across histological steatosis grades ($p=0.108$; Kruskall-Wallis test)

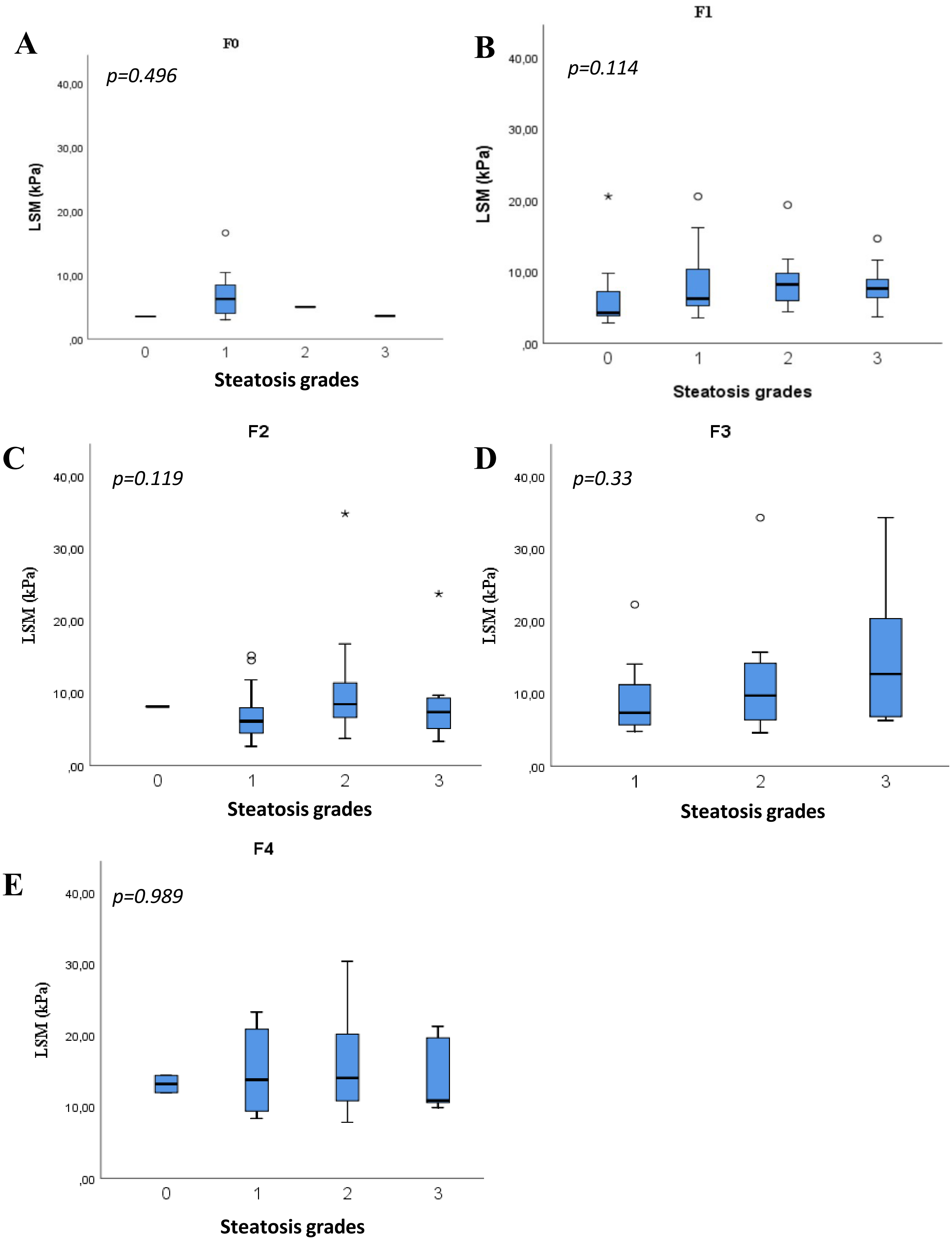

<u>Fig. S5:</u> LSM values distribution according to steatosis grades, within patients with F0 (A ; n=16), F1 (B ; n=75), F2 (C ; n=54), F3 (D ; n=26) and F4 (E ; n=37) (Kruskall-Wallis test)

A

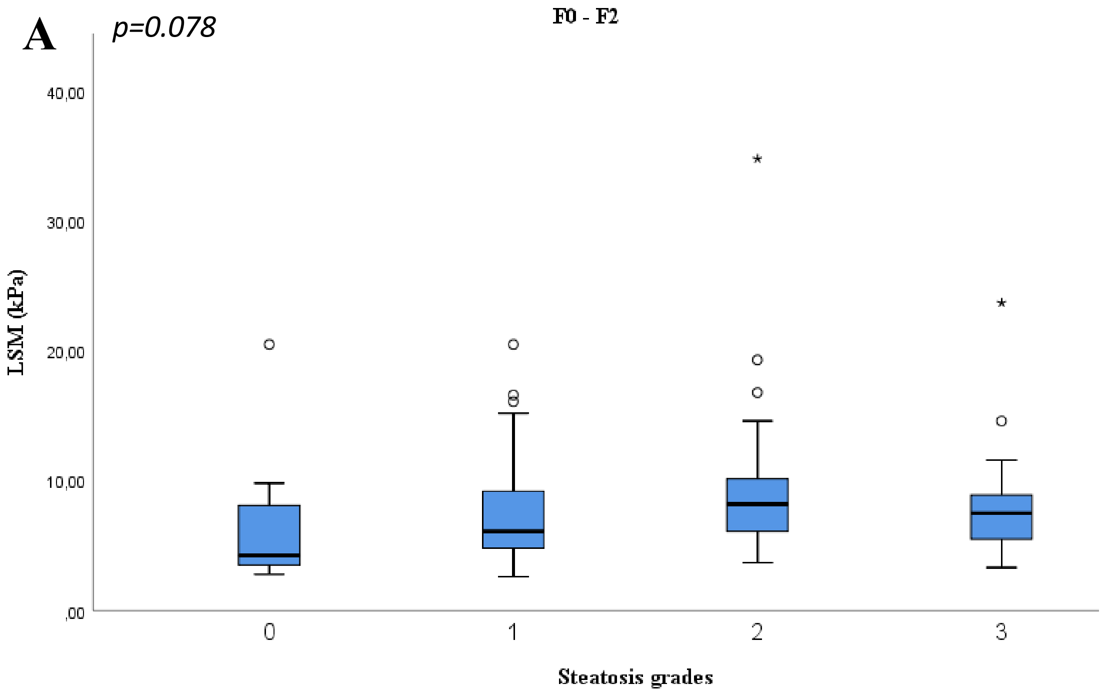

B

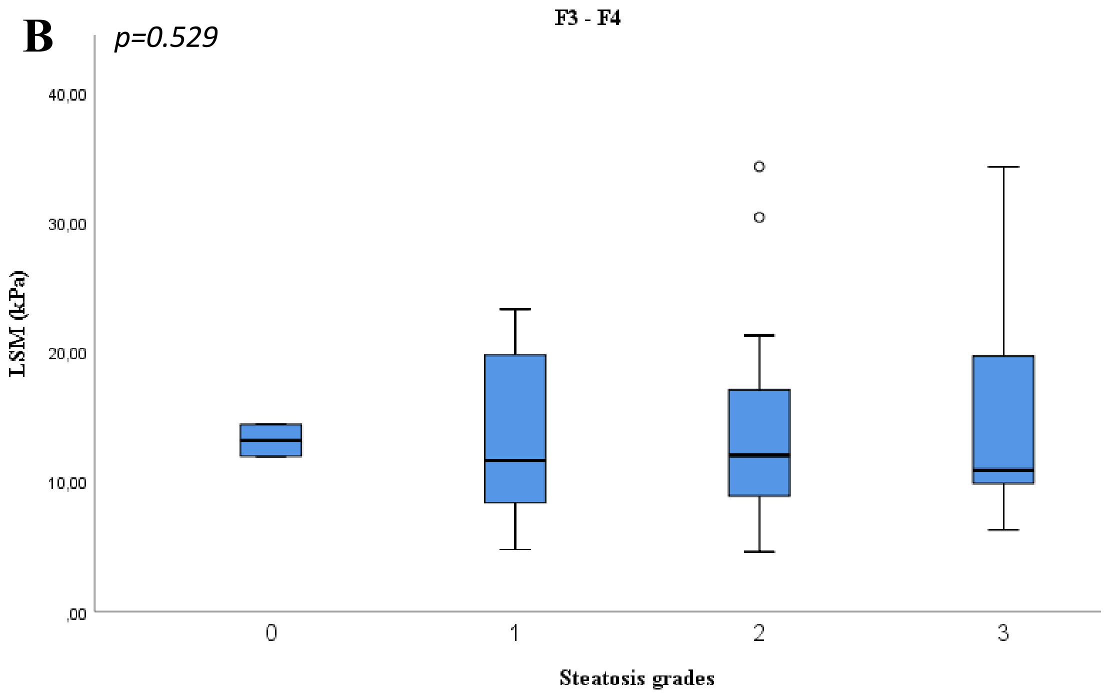

Fig. S6: LSM values distribution according to steatosis grades, within patients with F0-F2 fibrosis (A ; n= 145) and F3-F4 fibrosis (B ; n= 63) (Kruskall-Wallis test)

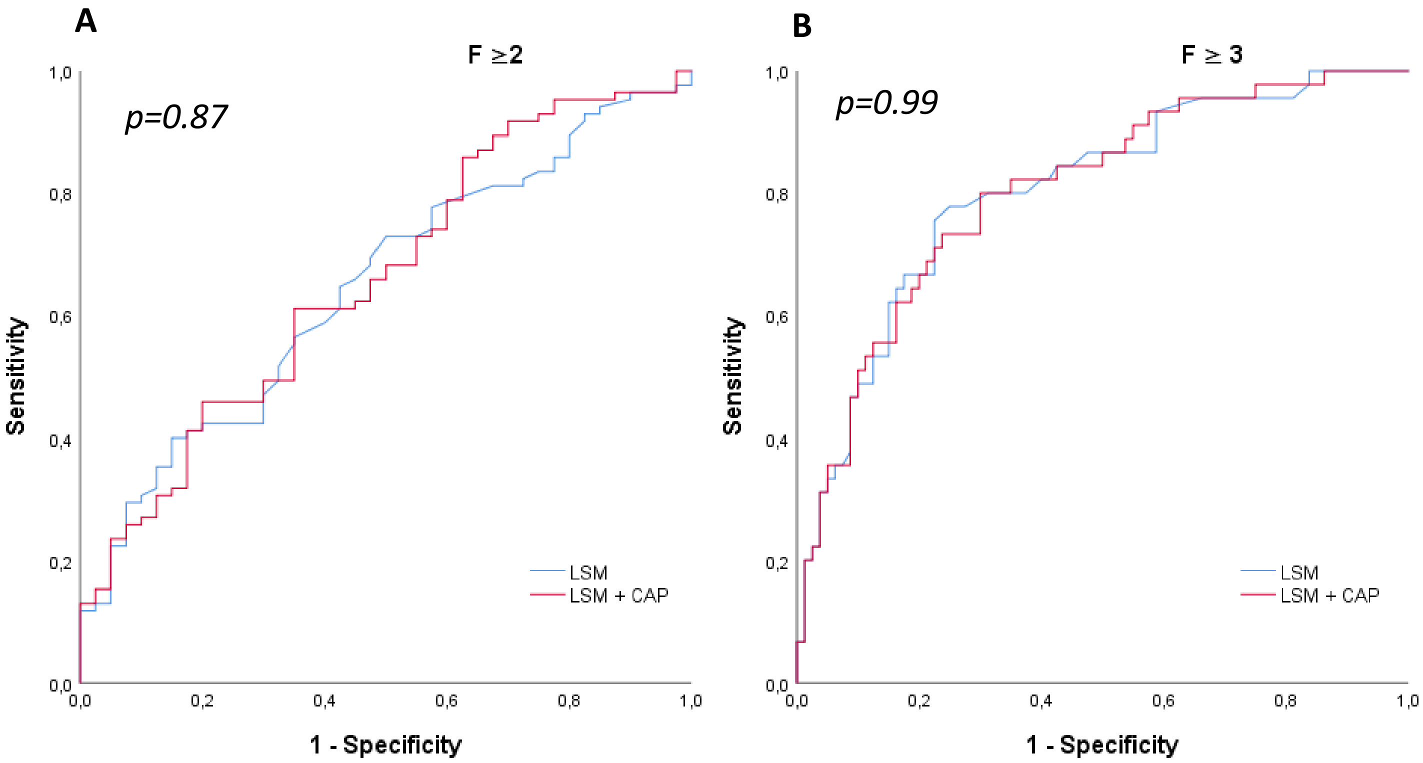

<u>Fig. S7:</u> AUROCs of LSM and LSM + CAP for diagnosing fibrosis stage ≥F2 (A) and ≥F3 (B) (Delong test)

Table S1: Accuracy of the 8kPa and 12 kPa cut off for ruling in or ruling out fibrosis stage ≥F3

Cutoff (kPa)	Sensitivity (%)	Specificity (%)	PPV (%)	NPV (%)
**8**	79.4	60.7	46.7	87.1
**12**	49.2	88.3	64.6	80

Table S2: Diagnostic accuracy of LSM and LSM+PDFF for each stage of fibrosis, according to diabetes status

	**AUROC [CI95%] LSM**			**AUROC [CI95%] LSM+PDFF**		
**Fibrosis stage**	*Diabetes*	*No diabetes*	*p-value*	*Diabetes*	*No diabetes*	*p-value*
**≥1**	0.50 [0.40-0.60]	0.65 [0.48-0.83]	0.132	0.84 [0.76-0.91]	0.69 [0.54-0.84]	0.075
**≥2**	0.69 [0.58-0.79]	0.63 [0.53-0.74]	0.48	0.66 [0.55-0.77]	0.64 [0.54-0.74]	0.80
**≥3**	0.74 [0.64-0.84]	0.80 [0.70-0.90]	0.43	0.76 [0.66-0.85]	0.78 [0.68-0.89]	0.75
**≥4**	0.81 [0.72-0.89]	0.86 [0.78-0.94]	0.39	0.79 [0.70-0.83]	0.79 [0.64-0.94]	0.99
**Fibrotic MASH**	0.72 [0.61-0.82]	0.3 [0.61-0.85]	0.86	0.72 [0.62-0.82]	0.72 [0.59-0.64]	0.96

p-values are those of AUROCs comparison using Delong test.

Table S3: Diagnostic accuracy of LSM and LSM+PDFF for each stage of fibrosis, according to body mass index

	**AUROC [CI95%] LSM**			**AUROC [CI95%] LSM+PDFF**		
**Fibrosis stage**	*BMI ≥30*	*BMI <30*	*p-value*	*BMI ≥30*	*BMI <30*	*p-value*
**≥1**	0.66 [0.38-0.93]	0.68 [0.49-0.87]	0.88	0.75 [0.55-0.91]	0.63 [0.41-0.85]	0.47
**≥2**	0.67 [0.57-0.76]	0.66 [0.54-0.78]	0.94	0.65 [0.56-0.75]	0.65 [0.53-0.77]	0.94
**≥3**	0.80 [0.72-0.87]	0.75 [0.63-0.87]	0.54	0.80 [0.72-0.88]	0.74 [0.61-0.87]	0.46
**≥4**	0.82 [0.74-0.89]	0.88 [0.79-0.97]	0.25	0.81 [0.72-0.89]	0.81 [0.64-0.98]	0.96
**Fibrotic MASH**	0.73 [0.63-0.82]	0.72 [0.63-0.82]	0.97	0.74 [0.65-0.83]	0.69 [0.5-0.85]	0.64

p-values are those of AUROCs comparison using Delong test.

Table S4 : Diagnostic accuracy of LSM and LSM+CAP

N=125	LSM		LSM + CAP		
**Fibrosis stage**	*AUROC*	*95%CI*	*AUROC*	*95%CI*	*p-value*
**≥2**	0.65	[0.55-0.75]	0.66	[0.56-0.76]	0.87
**≥3**	0.80	[0.72-0.88]	0.80	[0.72-0.88]	0.99
**Fibrotic MASH**	0.75	[0.66-0.84]	0.75	[0.66-0.84]	0.96

Because of missing data regarding CAP, AUROCs comparing LSM to LSM+CAP were calculated and compared in 125 patients.
